# Supplementary material for: Venomix: a simple bioinformatic pipeline for identifying and characterizing toxin gene candidates from transcriptomic data
Source: PeerJ. 2018 Jul 31;6:e5361. doi: 10.7717/peerj.5361 (PMC6074769; doi:10.7717/peerj.5361)
Supplement: Supplemental Information 3 [file peerj-06-5361-s003.gz › FinalOutput_E-6/Kunitz-type_serine_protease_inhibitor_conotoxin_Cal9/finaltree.pdf]

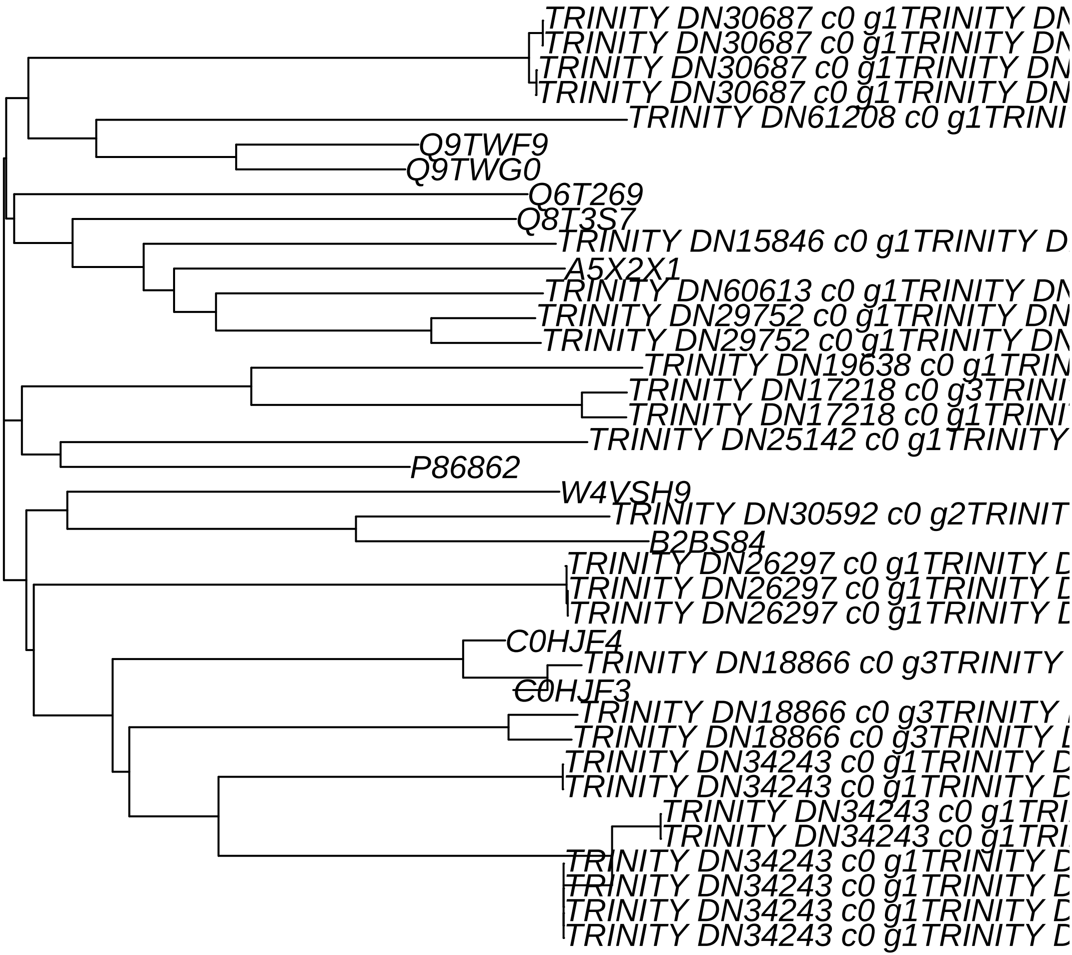
TRINITY DN30687 c0 g1TRINITY DN
TRINITY DN30687 c0 g1TRINITY DN
TRINITY DN30687 c0 g1TRINITY DN
TRINITY DN30687 c0 g1TRINITY DN
TRINITY DN61208 c0 g1TRINI
Q9TWF9
Q9TWG0
Q6T269
Q8T3S7
TRINITY DN15846 c0 g1TRINITY D
A5X2X1
TRINITY DN60613 c0 g1TRINITY DN
TRINITY DN29752 c0 g1TRINITY DN
TRINITY DN29752 c0 g1TRINITY DN
TRINITY DN19638 c0 g1TRIN
TRINITY DN17218 c0 g3TRINI
TRINITY DN17218 c0 g1TRINI
TRINITY DN25142 c0 g1TRINITY
P86862
W4VSH9
TRINITY DN30592 c0 g2TRINIT
B2BS84
TRINITY DN26297 c0 g1TRINITY D
TRINITY DN26297 c0 g1TRINITY D
TRINITY DN26297 c0 g1TRINITY D
C0HJF4
TRINITY DN18866 c0 g3TRINITY
C0HJF3
TRINITY DN18866 c0 g3TRINITY
TRINITY DN18866 c0 g3TRINITY D
TRINITY DN34243 c0 g1TRINITY D
TRINITY DN34243 c0 g1TRINITY D
TRINITY DN34243 c0 g1TRI
TRINITY DN34243 c0 g1TRI
TRINITY DN34243 c0 g1TRINITY D
TRINITY DN34243 c0 g1TRINITY D
TRINITY DN34243 c0 g1TRINITY D
TRINITY DN34243 c0 g1TRINITY D
